# Middle-aged and older adults in Aids village: a mixed methods study on talking about death and well-being promotion based on social support theory

**DOI:** 10.3389/fpsyg.2024.1363047

**Published:** 2024-03-20

**Authors:** Lin Zhang

**Affiliations:** Department of Journalism and Communication, Shanghai University, Shanghai, China

**Keywords:** social support, subjective well-being, talking about death, the HIV/Aids-affected middle-aged and older adults, mixed methods

## Abstract

**Introduction:**

Will about talking about death bring well-being? This study aims to explore the impact of talking about death on the subjective well-being of the rural middle-aged and older adults in China’s “Aids village” from five dimensions: the way of talking about death, the attitude towards talking about death, the objects of talking about death with, the frequency of talking about death and the content of the death talk, and investigate whether social support played a mediating role during this process.

**Methods:**

A field survey and in-depth interviews were conducted in Wen Lou village (a famous Chinese “Aids village”), and valid questionnaires were completed by the HIV/Aids-affected middle-aged and older adults. A series of linear regression analyses were conducted to detect whether the way of talking about death, the attitude towards talking about death, the objects of talking about death with, the frequency of talking about death and the content of the death talk predict the subjective well-being of the HIV/Aids-affected middle-aged and older adults. An empirical test for mediation effect was performed to examine whether social support played a mediating role during the process.

**Results:**

It was found that the more frequent the middle-aged and older adults talk about death, the higher level of their subjective well-being is (Δ*R*^2^ = 0.056, 0.05 < *p* < 0.10), and during which process social support played a mediating role.

**Discussion:**

The author believes that using “talking about death” as a kind of medical intervention, carrying out corresponding life education and death education, and developing a suitable hospice care model, may be a valuable way for the HIV/Aids-affected middle-aged and older adults in the rural area.

## 1 Introduction

The third Sustainable Development Goal (SDG-3) has a target to end the epidemic of HIV/Aids by 2030 (Project 2030). This will be achieved when the number of new HIV infections and “Aids-related deaths” decline by 90% between 2010 and 2030 (Assefa and Gilks, 2020). However, the number of middle-aged and older adults with HIV infection will continue to increase in the rural area, bringing new challenges globally (Godfrey et al., 2022). Aids prevention and control is high on China’s policy agenda, media agenda and public agenda, in contrast, the HIV/Aids-affected middle-aged and older adults have not got enough attention in academic realm. More than 90% of the research on Aids focus on children, ignoring the middle-aged and older adults. Additionally, research on Aids is often conducted from the perspectives of public health and government policies (Nsagha et al., 2015), with few from socio-cultural perspectives. Sociological barriers originate from the general intention to avoid topics such as death and dying in society (Seifart et al., 2020), thus little research has explored the relationships between talking about death and the subjective well-being of the HIV/Aids-affected middle-aged and older adults. However, it is proved that talking about death with families can provide emotional support (Mori et al., 2017). Social support has a positive predictive effect on subjective well-being among older adults of rural China (Su et al., 2022). Therefore, it is urgent and important to explore the relationships between talking about death and the subjective well-being among the HIV/Aids-affected middle-aged and older adults. A large number of HIV/Aids-affected middle-aged and older adults are distributed in the rural areas. However, most academic research focus on the urban HIV/Aids-affected middle-aged and older adults, and research on the groups in the rural area is almost blank.

Wen Lou village, used to be the most densely HIV-infected area in China in the 1990s (Zhang, 2023). There were more than 3,000 villagers in the village in the past, and 70% of them were infected by HIV, making the village a well-known “Aids village” globally. Li Keqiang, Wen Jiabao, Wu Yi and other Chinese national leaders have inspected there many times. At present, there are 343 villagers with HIV infection living in Wen Lou, including 331 current patients, among whom 270 patients take antiviral drugs. Most of the 343 villagers are over 50 years old. In August 2021, we conducted a field survey and performed in-depth interviews with the HIV/Aids-affected middle-aged and older adults in Wen Lou village. 42 valid questionnaires were collected and face-to-face in-depth interviews with 32 middle-aged and older adults were performed.

## 2 Literature review

### 2.1 Social support theory

Cobb (1976) raises that social support includes three dimensions: feeling loved, feeling valued or respected, and belonging to a social network. House and Khan (1985) regard social support as the functions performed for the individual by significant others, such as family members, friends, coworkers, relatives, and neighbors, etc., which typically include social and emotional assistance, practical assistance, and informational assistance. Some scholars believe that social support includes emotional support, instrumental support, informational support and appraisal support (House, 1983; Barrera, 1986; Tilden and Weinert, 1987). Barrera and Ainlay (1983) divide social support into six categories: material aid, such as practical assistance with money and other materials; behavioral assistance, such as work that shares physical labor; intimate interaction behavior, such as listening, showing respect, care, understanding, etc.; guidance, such as providing help, information and guidance; feedback, such as providing personal feedback about their behaviors, thoughts and feelings; positive social interaction, such as participating in entertainment and relaxing social interaction. Regarding with domestic research, Haixiong et al. (1998) believe that social support involves both internal and external support and maintenance of the family, as well as various formal and informal support and assistance. Social support is not just a one-way care or help, it is a social exchange in most cases. Zhang and Danqing (1999) claim that “social support refers to the various help people obtain from society and from others.” Can (2020) point out that social support can be divided into objective support and subjective support, or actual social support and perceived social support. Objective support or actual social support includes material assistance, direct service etc. received by individuals. Subjective support or perceived social support refers to the emotional experience of being respected, understood, and supported felt by individuals.

### 2.2 Subjective well-being

Subjective well-being (SWB) is a state in which every individual realizes his or her own potential, can cope with the normal stresses of life, can work productively and fruitfully, and is able to make a contribution to her or his community (Booth et al., 2021). SWB is an important indicator of the level of mental health and quality of life of the middle-aged and older adults, which is closely related to life satisfaction, happiness and social inclusion (Vilkhu and Behera, 2019). The literature on SWB is vast and cover multiple disciplines, focusing on diverse determinants and correlates of SWB, ranging from demographics, personality, geography, and supportive relationships to health status. Factors potentially influencing personal orientation, fulfillment and engagement, evaluations, and emotions are considered determinants/correlates of SWB, which fall into seven broad categories: (1) basic demographics, (2) socioeconomic status, (3) health and functioning, (4) personality, (5) social support, (6) religion and culture, (7) geography and infrastructure (Das et al., 2020). In addition, the role and quality of neighborhood services, social capital and social cohesion are significantly associated with the SWB of Chinese middle-aged and older adults (Clark et al., 2019).

### 2.3 Social support and SWB

A large body of research has consistently found a positive association between social support and SWB across a variety of mental health outcomes (Chu et al., 2010). For example, greater social support is associated with more positive feelings and life satisfaction, as well as less depression, anxiety, and stress (Siedlecki et al., 2014; Klainin-Yobas et al., 2016). People with high levels of social support have higher levels of health and SWB (Wang, 2016). Furthermore, social support has a positive predictive effect on the SWB among middle-aged and older adults of rural China (Su et al., 2022). Social support (emotional support, information access, companionship, financial support) has significant effect on the SWB of the middle-aged and older adults in Nigeria (Oluwagbemiga, 2016).

### 2.4 Talking about death

Death is a taboo in traditional values (Tu et al., 2022). People usually avoid talking about death with the middle-aged and older adults in their lives. However, being able to talk about death with others freely has an important impact on the SWB of the middle-aged and older adults. For example, nursing staff discussing the topic of death with the middle-aged and older adults and their families can provide them with spiritual comfort and psychological counseling, so that the middle-aged and older adults can correctly understand death and free themselves from fear (Chua and Shorey, 2021). Overall, prior findings suggest that the “taboo” against death talk may be more perceived than real (Wilson et al., 2022). We will review the literature on talking about death from five dimensions: the way of talking about death, the attitude toward talking about death, the objects of talking about death with, the frequency of talking about death and the content of the death talk.

#### 2.4.1 The way of talking about death

Generally speaking, the more actively people talk about death, the more positive their attitudes toward death will be. Being able to regard death as a topic in daily conversation shows that an individual have a normal attitude toward death. Whether an individual can take the initiative to talk about death reflects the degree of identification with death avoidance. Some scholars believe that the middle-aged and older adults with a low degree of identification have a higher SWB, because they regard death as part of the life process, and treat death with a normal mentality without fear or avoidance. The middle-aged and older adults with a high degree of identification also have a higher level of SWB, because they fear death, unwilling to involve anything related to death, or even unwilling to hear “death,” reject consciously all information related to death out of consciousness. But the middle-aged and older adults with a moderate degree of identification have a lower level of SWB, because they have not yet formed a scientific understanding of death and are afraid of death. There are often some death-related things in their brains, but they cannot control not to think about them, so they are miserable (Liu et al., 2013). The social support that the middle-aged and older adults accept in their lives, whether it comes from within or outside the family, will raise the middle-aged and older adults’ awareness of death. Because social support will bring more information and knowledge to people, and by communicating with others, the middle-aged and older adults will have a more rational perception of life and death. The higher the social support of the middle-aged and older adults obtain, the more positive their attitudes toward death. Therefore, the hypotheses are proposed:

*H1*: (a) The more actively the middle-aged and older adults talk about death, the higher level of their SWB, (b) and during which process social support plays a mediating role.

#### 2.4.2 The attitude toward talking about death

The attitudes toward talking about death are optimistic or pessimistic of the middle-aged and older adults, will affect their SWB. An empirical study by Liu et al. (2013) on the attitudes toward death among urban middle-aged and older adults showed that the more positively and optimistically the middle-aged and older adults who face death, the happier and peaceful they are. On the contrary, when the middle-aged and older adults in nursing homes face death, most of them will experience fear, anxiety and even escape and other pessimistic emotions. Because without the company of families or relatives, the negative feelings of the middle-aged and older adults cannot be vented and dismissed, which in turn affect their SWB (Sun et al., 2011). However, social support plays an important role in improving the SWB of the middle-aged and older adults by giving them a higher sense of security, and people with strong social support will have a lower fear of death. There is a significant negative correlation between social support and death fear (Bibi and Khalid, 2020). In addition, the middle-aged and older adults with more social support have more interactions with people, and the information and knowledge they obtain from others correspondingly increase, which improves their understanding of the nature of life, thereby reducing fear of death and improving SWB (Zhou et al., 2013). Therefore, the hypotheses are proposed:

*H2*: (a) The more optimistically the middle-aged and older adults talk about death, the higher level of their SWB, (b) and during which process social support plays a mediating role.

#### 2.4.3 The objects of talking about death with

The objects of talking about death with can be divided into self-speaking, primary groups (such as family), secondary groups (such as relatives and friends), and casual groups. The middle-aged and older adults who talk about death with family members, relatives and friends, or casual groups, have a positive attitude toward death. While the middle-aged and older adults who only communicate with themselves about death, have a more negative attitude toward death. Because as mentioned above, the middle-aged and older adults who can deal with death in a normal mind are more able to talk about death more freely. The middle-aged and older adults who are not fully aware of death because of fear, are reluctant to mention or even cannot listen to the topic of death (Jing et al., 2012). Social activities between the middle-aged and older adults and families, relatives and friends have an important impact on SWB, and may play a role in buffering the negative effects of aging (Huxhold et al., 2014). Studies have confirmed that the more objects the middle-aged and older adults socialize with, the higher the level of SWB (Qiao et al., 2018). Therefore, the hypotheses are proposed:

*H3*: (a) The more objects the middle-aged and older adults talk about death with, the higher level of their SWB, (b) and during which process social support plays a mediating role.

#### 2.4.4 The frequency of talking about death

Similar to the way of talking about death and the attitude toward talking about death, the frequency of talking about death reflects how the middle-aged and older adults view death. The middle-aged and older adults who try to avoid talking about death with others have low acceptance of death and a higher fear of death (Zheng et al., 2014). The fear of death is negatively correlated with SWB (Gesser et al., 1988). Additionally, the middle-aged and older adults with low social support have a higher fear of death, a more negative attitude toward death, and therefore a lower level of SWB. On the contrary, the higher frequency of talking about death in daily life in normalizing death and bringing it into our “everyday” conversation, the stronger people feel they would be better equipped to deal with loss and fear of death more effectively. Because “talking” is recognized as a two-way interaction and people get more social support from others during more frequent two-way interactions (Wilson et al., 2022). Therefore, the hypotheses are proposed:

*H4*: (a) The higher frequency the middle-aged and older adults talk about death, the higher level of their SWB, (b) and during which process social support plays a mediating role.

#### 2.4.5 The content of the death talk

**Figure 1:**
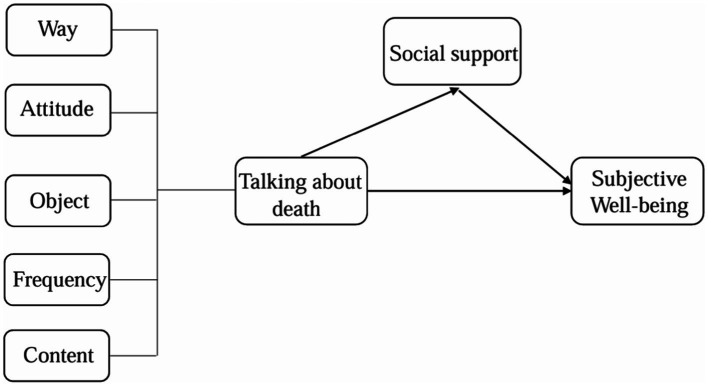
Proposed research model. Way, the way of talking about death; Attitude, the attitude toward talking about death; Object, the objects of talking about death with; Frequency, the frequency of talking about death; Content, the content of the death talk.

The content of the death talk mainly includes the eschatology and the afterlife. The eschatology believes that life has come to an end, and there is no afterlife. The afterlife is a saying in religion that believes after this life is over, there will be another life. This religious belief is a major factor influencing the middle-aged and older adults’ fear of death, and the middle-aged and older adults with high level of religious belief have relatively low level of fear of death (Cicirelli, 2011). Pursuing afterlife or even immortality is an imagination beyond death, which reflects the anxiety of the middle-aged and older adults about death. At present, the traditional mentality of “tending to live and avoid death” still exists among the middle-aged and older adults in urban communities. Some of the middle-aged and older adults accept death as “a last resort” and passively accept death (Liu et al., 2012). The rural middle-aged and older adults whose mental status is optimistic or normal are more positive or calm about death than those whose mental status is fearful or anxious (Bidzan-Bluma et al., 2020). The middle-aged and older adults with poorer mental function are more likely to be pessimistic about real life and regard death as a beautiful afterlife, or as a way to escape the miserable life (Huang and Lin, 2004). The perceived high level of social support is associated with positive mental and psychological health outcomes (Li et al., 2021). The quality and structure of social networks are important for maintaining mental health (Newman and Zainal, 2020), and support in the form of encouragement, consideration and conversation helps the middle-aged and older adults to obtain a kind of psycho-social support, which can significantly improve the mental health and SWB of the middle-aged and older adults (Putran et al., 2022). Moreover, mental health plays a mediating role between social support and life satisfaction of the middle-aged and older adults (Xiaomin, 2016). Therefore, the hypotheses are proposed:

*H5*: (a) The more attention paid to the afterlife when the middle-aged and older adults talk about death, the lower level of their SWB, (b) and during which process social support plays a mediating role (Figure 1).

## 3 Methods

### 3.1 Procedure and sample

We went to Wen Lou village and conducted a field survey in August 2021. All of the local villagers with registered residence above 50 years old living in the village were invited to participate in this study, others were excluded. Some refused us by the reasons such as hard of hearing, or difficulties in understanding the questions. Printed questionnaires were distributed to the participants. Each questionnaire took about 15–20 min. For those illiterate participants, we read the questions and items to them firstly, then they told us the answers, and last we helped them to complete the questionnaires. The questionnaires were collected as soon as they were completed. Once the participants finished the questionnaires, they were debriefed and thanked. After eliminating incomplete data, 42 valid questionnaires were analyzed. All participants received the information about the purpose of the research and signed the consent forms.

This study was approved by the Internal Review Board of Shanghai Jiao Tong University (no. H2021198I), which ensured that the research complied with ethical standards and safeguards the rights and privacy of the participants.

### 3.2 Interviews

After the questionnaire survey, we conducted face-to-face in-depth interviews with 32 HIV/Aids-affected middle-aged and older adults. Each interview was completed with an average duration of approximately 1–1.5 h, thus each participant was available to provide reliable accounts of their experiences. During the interviews, the researchers asked each participant 17 open-ended questions, such as “Would you actively talk about the topic of death with your family and why?” “Do you have any thoughts and plans for the future?” and so forth. Follow-up questions also were included to clarify issues and to validate researcher interpretations. The participants were informed that the in-depth interviews would be tape-recorded for data analysis later.

### 3.3 Data analysis

The participants’ accounts of their experiences in the interviews were transcribed into English texts by the researchers. The questionnaire data were analyzed using SPSS-13 software by descriptive statistics, and a series of linear regression analyses and mediation analyses.

### 3.4 Measures

To ensure that the instruments used in this study are valid for the middle-aged and older adults, we used previously validated scales to measure all the key variables, and adhered to the licensing requirements which the authors have specified.

*Social support* was measured by 8 items adapted from the “Social Support Rating Scale” proposed by Xiao and Yang (1987). The original scale has 3 dimensions and 10 items, and we deleted 2 items irrelevantly and kept 8 items. The three dimensions are: (1) subjective support: the degree of personal self-perception of the support of others; (2) objective support: the number of personal connections with others; (3) personal utilization: when the individual encounters life events, the degree to which he can use the support and help of others. The higher the score of the social support rating scale, the more social support the participants receive. The Cronbach’s alpha was.90.

*Talking about death* was measured by 5 dimensions including the way of talking about death, the attitude toward talking about death, the objects of talking about death with, the frequency of talking about death and the content of the death talk with 10 items. All items were responded to on a 5-point Likert Scale ranging from “1 = strongly disagree” to “5 = strongly agree.” The Cronbach’s alpha was 0.89.

*Subjective well-being* was measured using “Revision of the positive affect and negative affect scale” developed by Lin et al. (2008) which contains 4 dimensions and a total of 24 items, specifically, a total of 10 items in the positive and negative affective dimensions, and a total of 14 items in the positive and negative experience dimensions. Among them, choose “Yes” to count 1 point, and choose “No” to count 0 point. The total score of the scale = positive affect score-negative affect score + positive experience score-negative experience score + 24. The total score of the scale ranges from 0 to 48. The higher the score, the higher level of the individual’s SWB. In this study, the internal consistency coefficients of each dimension of the scale are 0.81 (positive affect), 0.64 (negative affect), 0.59 (positive experience), and 0.67 (negative experience), respectively. The Cronbach’s alpha was 0.85.

*Demographic variables*, consisting of sex, age, ethnic, marital status, residence, education level, income, religion and whether the participant or his/her families/relatives were with HIV infection were measured as controlling variables.

## 4 Results

### 4.1 Descriptive results

After eliminating incomplete data, 42 participants provided valid questionnaires. The demographic characteristics of the 42 participants are presented in Table 1. Among the 42 participants, the level of social support was high (*M* = 0.8973, SD = 0.21621). The level of attitude toward death was moderate (*M* = 3.4656, SD = 0.46448). Regarding with talking about death, the level of the way of talking about death was moderate (*M* = 3.2857, SD = 0.90488), the level of the attitude toward talking about death was rather high (*M* = 4.095, SD = 1.0075), the level of the objects of talking about death with was moderate (*M* = 2.5381, SD = 0.97329), the level of the frequency of talking about death was low (*M* = 2.048, SD = 1.4134), the level of the content of the death talk was considerably high (*M* = 4.429, SD = 1.1507). The subjective well-being was at a moderate level (*M* = 22.1905, SD = 4.68645).

**Table 1:** Sociodemographic characteristics of the participants.

Sociodemographic characteristics	Responses	Percentage
Gender		
Female	22	52.38%
Male	20	47.62%
Age		
50–59	12	28.6%
60–69	15	35.7%
70–79	11	26.2%
80–89	4	9.5%
Individual Health Status		
Healthy	13	30.95%
HIV carriers and have no other disease	5	11.9%
Without HIV infection but have other disease	17	40.48%
Aids patients and have other disease	7	16.67%
Family’s Health Status		
With HIV infection families	15	35.71%
Without HIV infection families	27	64.29%
Living Status		
Live with spouse and the next generation	3	7.14%
Live with spouse	15	35.71%
Live with the next generation	9	21.43%
Live alone	8	19.05%
Live in Xingfu Yuan (Nursing home)	1	2.38%
Religious faith		
Yes	4	9.52%
No	38	90.48%
Education		
Illiteracy	18	42.86%
Primary school	13	30.95%
Middle school	10	23.81%
High school or technical secondary school	1	2.38%

### 4.2 Hypothesis testing

Linear regression analyses were conducted to detect the relationships between the way of talking about death, the attitude toward talking about death, the objects of talking about death with, the frequency of talking about death and the content of the death talk and the subjective well-being of the participants. H1a (see Table 2), H2a (see Table 3), H3a (see Table 4) and H5a (see Table 5) were all not supported, but H4a (see Table 6) was marginally supported.

**Table 2:** The regression analysis of the way of talking about death.

Predictors	*β*	Δ*R*^2^
Block 1: Demographic variables		0.318
Gender	−0.542**	
Age	−0.222	
Marital	0.169	
Education	−0.421*	
Residence	−0.203	
Block 2		0.010
The way of talking about death	0.116	
	Adjusted *R*^2^ = 0.213	

*p < 0.05, **p < 0.01, ***p < 0.001.

**Table 3:** The regression analysis of the attitude toward talking about death.

Predictors	*β*	Δ*R*^2^
Block 1: Demographic variables		0.318
Gender	−0.522**	
Age	−0.287^#^	
Marital	0.214	
Education	−0.408*	
Residence	−0.176	
Block 2		0.041
The attitude toward talking about death	0.214	
	Adjusted *R*^2^ = 0.249	

^#^0.05 < *p* < 0.10, *p < 0.05, **p < 0.01, ***p < 0.001.

**Table 4:** The regression analysis of the objects of talking about death with.

Predictors	*β*	Δ*R*^2^
Block 1: Demographic variables		0.318
Gender	−0.538**	
Age	−0.219	
Marital	0.179	
Education	−0.432*	
Residence	−0.188	
Block 2		0.005
The objects of talking about death with	0.081	
	Adjusted *R*^2^ = 0.207	

*p < 0.05, **p < 0.01, ***p < 0.001.

**Table 5:** The regression analysis of the content of the death talk.

Predictors	*β*	Δ*R*^2^
Block 1: Demographic variables		0.318
Gender	−0.572***	
Age	−0.260^#^	
Marital	0.203	
Education	−0.496*	
Residence	−0.195	
Block 2		0.003
The content of the death talk	−0.072	
	Adjusted *R*^2^ = 0.205	

^#^0.05 < *p* < 0.10, *p < 0.05, **p < 0.01, ***p < 0.001.

**Table 6:** The regression analysis of the frequency of talking about death.

Predictors	*β*	Δ*R*^2^
Block 1: Demographic variables		0.318
Gender	−0.480**	
Age	−0.163	
Marital	0.119	
Education	−0.397*	
Residence	−0.122	
Block 2		0.056
The frequency of talking about death	0.265^#^	
	Adjusted *R*^2^ = 0.266	

^#^0.05 < *p* < 0.10, *p < 0.05, **p < 0.01, ***p < 0.001.

**Figure 2:**
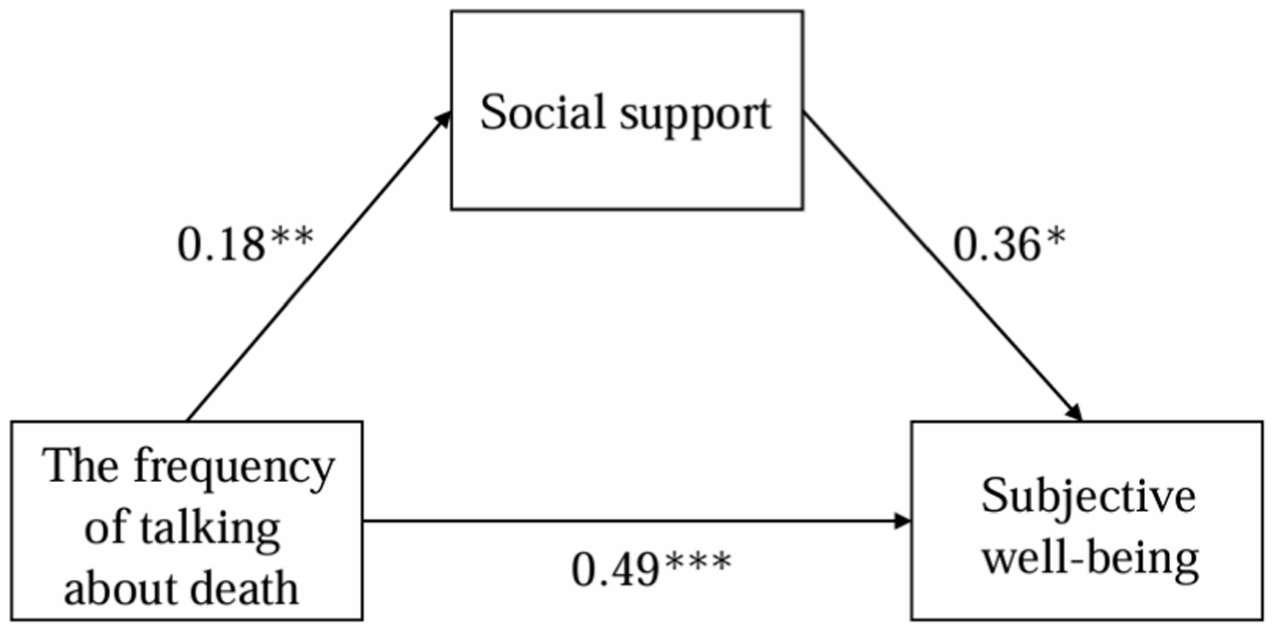
The relationship model of the frequency of talking about death, social support and subjective well-being.

Mediation analyses were conducted to detect H1b, H2b, H3b, H4b, and H5b. H1b, H2b, H3b, and H5b were not supported, but H4b was supported (see Table 7 and Figure 2).

**Table 7:** Total effect, direct effect and indirect effect of the frequency of talking about death on subjective well-being.

	Effect	Boot SE	95%CI		Percentage
			Boot LLCI	Boot ULCI	
Total Effect	1.6088	0.4762	0.4248	2.3496	/
Direct Effect	1.3872	0.4509	0.6968	2.5208	86.2%
Indirect Effect	0.2216	0.2668	0.3264	0.7526	13.8%

## 5 Discussion

The idea that the public has an aversion to talking about death and dying, and that death remains a social ‘taboo’ has been vigorously promoted by a diverse range of social movements and interest groups since the 1960s. Nevertheless, then as now, there is little evidence to support these claims as attitudes to death and dying have not been extensively researched (Wilson et al., 2022). What’s more, there were little research on the relationships between talking about death and subjective well-being among the middle-aged and older adults. In response to these gaps, this study explored the impacts of the way of talking about death, the attitude toward talking about death, the objects of talking about death with, the frequency of talking about death and the content of the death talk on the subjective well-being of the HIV/Aids-affected middle-aged and older adults, and investigated whether social support played a mediating role during this process. Partly in line with prior research which found that talking about personal death or loss is a powerful way of healing the self, improving well-being as well as strengthening the potential for individuals to support each other (Kellehear, 2012: ix), our findings illustrate that the higher frequency the middle-aged and older adults talk about death, the higher level of their SWB, and during which process social support plays a mediating role. However, the way of talking about death, the attitude toward talking about death, the object of talking about death and the content of the death talk could not predict a higher level of SWB, nor during which process social support plays a mediating role, which are inconsistent with previous research. These findings emphasize that a high frequency of talking about death is positively correlated with a high level of SWB of the HIV/Aids-affected middle-aged and older adults, while talking about death actively or not, talking about death optimistically or not, talking to more people about death or not, and talking about the afterlife or eschatology seem not to be related to the SWB of the HIV/Aids-affected middle-aged and older adults significantly.

Why the higher frequency the middle-aged and older adults talk about death, the higher level of their SWB? We can interpret this finding from the in-depth interviews of the participants. Death is a taboo topic in traditional ideas, however, talking about death frequently means regarding death as a part of everyday life and death might be “normalized.” After a “normalization of death,” death will be more visible and discussed like many elements of life. Additionally, there is good evidence that honest discussion of difficult information may enable people to feel more empowered about care and decision-making, and enhance rather than diminish hope (Davison and Simpson, 2006).

While, why talking about death actively or not, talking about death optimistically or not, talking to more people about death or not, and talking about the afterlife or eschatology were not related to the SWB of the HIV/Aids-affected middle-aged and older adults significantly? We can borrow some theories from ecological systems theory (Bronfenbrenner, 1979), which states that the SWB of middle-aged and older adults in their later years is closely related to their perception of environmental risks, and emphasizes the sustained and interactive stable role/identity relationship between humans and the environment. As follows:

“I usually actively talk about the topic of death in daily life, and I do not shy away from it. What makes me helpless is not death, but I am an Aids patient.” (A, male, with HIV infection, 70 years old).

“Aids patient” is the participants’ common identity, and the continuity of lifestyle and identity can affect life and well-being in old age (Atchley, 1989). Therefore, the SWB of the HIV/Aids-affected middle-aged and older adults is more determined by their identity compared with talking about death actively or not.

“I believe in fatalism. Birth, aging, illness and death are all arranged by fate. No matter an individual talk about death optimistically or pessimistically, if he/she gets sick and cannot live anymore, there is nothing he/she can do.” (B, male, with HIV infection, 62 years old).

Like participant B said, many participants in this study claimed that they believed in fatalism. Fatalism is a cognitive schema defined by passive and submissive acceptance of an irremediable destiny and feelings of hopelessness (Díaz et al., 2015). Previous research supported that a lower fatalism was associated with greater protective behaviors, and the lower the fatalism was, the higher the well-being was Caballero et al. (2022). Maybe that is the reason why talking about death optimistically or pessimistically is not related to SWB significantly.

“I do not like to talk about death with others, because I think it will make them feel uncomfortable and bring burden to them.” (C, male, with HIV infection, 55 years old).

“An individual’s death has nothing to do with the other people, because everyone is busy with himself. If an individual has a good mindset, he/she will feel peaceful and can die later. If an individual gets sick, he/she should fight against the illness.” (D, female, with HIV infection, 53 years old).

Prior research proved that perception of relevance to others was positively related to disclosure efficacy, and disclosure efficacy was positively related to intention to disclose death plan decisions and people’s SWB (Kuchenbecker and Bevan, 2023). In the views of most participants in this study, their lives have nothing to do with others, especially those not their family members, therefore, they are reluctant to talk about death with others. This may be why talking to more people about death or not is not related to their SWB significantly.

“I do not want to mention my past experience of HIV infection, nor do I think about my future plans and arrangements after my death. I live in the moment.” (E, female, with HIV infection, 58 years old).

“I prefer to talk about the present rather than the afterlife, which makes me feel more relaxed and pleased.” (F, male, with HIV infection, 63 years old).

Afterlife and eschatological beliefs can potentially reduce the existential threat of mortality. Specifically, belief in an afterlife can help to reduce anxiety around the prospect of death and comfort following bereavement (Stauner et al., 2020). Meanwhile, it is also proved that “live in the moment” and participate in physical activities actively is a highly contributory factor to people’s overall well-being (Sumption and Burnett, 2021). Maybe under the impact of participation in physical activities, talking about the afterlife or eschatology are not correlated to SWB significantly.

To conclude, death is in reality part of everyday life, and people should be encouraged to reflect on and talk about it, particularly in a supportive environment (Costin, 2015). The findings from the current investigation provide evidence that “talking about death frequently” may be an effective approach to improve the SWB of the HIV/Aids-affected middle-aged and older adults in the rural area from a socio-cultural and social supportive perspective. However, talking about death actively or not, talking about death optimistically or not, talking about death to more people or not, and talking about the afterlife or eschatology were not related to the SWB of the HIV/Aids-affected middle-aged and older adults in the rural area. This study contributes to a small but emergent research area examining the impacts of talking about death on the SWB among of the HIV/Aids-affected middle-aged and older adults in the rural area. Findings from this study demonstrated the potential benefits of an approach in tackling the taboos around death and dying, which can be used to inform new public health programs toward empowering the middle-aged and older adults to have these conversations with others in their community and families toward upstreaming advance care planning (ACP).

Several limitations should be noticed in the current study. First, the results may not be generalizable due to the small and potentially non-representative sample, therefore we discussed more based on the findings from the in-depth interviews to support the statistical analysis. Future research should be performed with a larger sample size. Second, the causality among the variables cannot be identified with this cross-sectional survey. To determine causality, longitudinal studies with panel data are needed to affirm the temporal order in the future.

## 6 Conclusion

There is greater recognition of the need for closer integration with mental health services and support for middle-aged and older adults with HIV (UNAIDS Global AIDS Update, 2023). This study confirmed that “talking about death frequently” was positively related to the subjective well-being of the HIV/Aids-affected middle-aged and older adults in the rural area, and social support played a mediating role during this process. Theoretically, this research adds a crucial angle to the research on “death taboo” among the key middle-aged and older population, and illustrates the positive influence of talking frequently about death on the subjective well-being of the middle-aged and older adults. Practically, this study shed light on a possible medical treatment to promote the subjective well-being of the HIV/Aids-affected middle-aged and older adults, and mitigate the impact of HIV/Aids on communities and societies.

## Data availability statement

The original contributions presented in the study are included in the article/supplementary material, further inquiries can be directed to the corresponding author.

## Ethics statement

The studies involving humans were approved by the review board of Shanghai Jiao Tong University. The studies were conducted in accordance with the local legislation and institutional requirements. Written informed consent for participation in this study was provided by the participants’ legal guardians/next of kin.

## Author contributions

LZ: Conceptualization, Data curation, Formal analysis, Funding acquisition, Investigation, Methodology, Project administration, Resources, Software, Supervision, Validation, Visualization, Writing – original draft, Writing – review & editing.
